# Editorial: Ecology and molecular biology of bloom-forming cyanobacteria

**DOI:** 10.3389/fmicb.2023.1346581

**Published:** 2023-12-12

**Authors:** George S. Bullerjahn, R. Michael L. McKay, Petra M. Visser

**Affiliations:** ^1^Department of Biology and Great Lakes Center for Fresh Waters and Human Health, Bowling Green State University, Bowling Green, OH, United States; ^2^Great Lakes Institute for Environmental Research, University of Windsor, Windsor, ON, Canada; ^3^Department of Freshwater and Marine Ecology, Institute for Biodiversity and Ecosystem Dynamics, University of Amsterdam, Amsterdam, Netherlands

**Keywords:** cyanobacteria, cyanotoxins, microcystins, secondary metabolites, harmful algal blooms

Given the worldwide expansion of cyanobacterial harmful algal blooms (cHABs), this Research Topic seeks to highlight the threats that bloom events pose to environmental and human health. As many studies link severity of cHABs to a warming climate and changing precipitation patterns, such events are expected to grow, with cHABs increasing in distribution, duration and frequency, contributing to unbalanced and degraded ecosystems. Addressing this global concern, research is aimed at understanding the environmental factors contributing to bloom formation and decline, toxin and metabolite synthesis, bloom ecology, and cHAB mitigation. This Research Topics collection of papers, largely drawn from contributions to the 12th International Conference on Toxic Cyanobacteria, aims to showcase the state of the science related to cHABs, which may point to potential solutions to this growing threat to our freshwater resources.

Several papers contributed to the Research Topic addressed community structure and the ecology of blooms. Whereas colonial *Microcystis* has become synonymous with blooms, there is increased recognition of community diversity between bloom locations and even within a bloom itself. In part, this is attributed to more widespread adoption of metabarcoding and genomics approaches as evidenced by each of the studies highlighted here. Beyond methodological advances, community structure often reflects environmental gradients to which communities are exposed. One such gradient is the fluvial-lacustrine continuum. Surveying bloom-forming regions of the Laurentian Great Lakes, Crevecoeur et al. identified a clear shift in dominance of the cyanobacterial communities between river and lake, yet also noted strong connectivity and dispersal of taxa within the system as a whole. Elsewhere, seasonality can play a role in shaping community structure. In Florida's Lake Okeechobee, Lefler et al. showed that cyanobacterial communities within the lake are significantly different between the wet and dry seasons. Also noted from this study was that the dominant cyanobacterial bloom formers in this lake maintain distinct bacterial associations that may confer competitive advantages across spatial and temporal scales.

In many cases, underlying environmental gradients are nutrients, the parameter conventionally recognized as dominant in the “bottom-up” control of cHABs. The important role of nutrients in shaping community structure was assessed in several of the contributing articles. Chen et al. frame the role of nutrients in a management framework, noting that closed-lake management practices adopted to control nutrient loading in the Taihu Basin River Network were successful at mitigating blooms of *Microcystis*. Using a metagenomic approach to compare the taxonomic and functional structure of the bacterioplankton community between open and closed lakes, differences were detected at fine taxonomic resolution (genus and species), but not extending to functional genes maintained by keystone taxa. In another managed system, Feng et al. assessed seasonal variability of microbial community composition in a subtropical reservoir following completion of a multi-year ecological restoration project aimed to reduce nutrient loading from adjacent tributaries. Their study showed negligible variability in community structure related to seasonality; however, the surveys showed enrichment of cyanobacterial communities in benthic samples pointing to possible internal loading as a nutrient source. Finally, Kim et al. examined microbial community structure and the presence of cyanotoxins in aerosols generated from wind-driven wave action in South Korea's Nakdong River. There is increased recognition of the potential threat of aerosols as a vector for cyanotoxin exposure, a concern reinforced by this study that showed toxigenic *Microcystis* to be one of the dominant genera in the aerosol microbiome.

Three articles in this Research Topic investigated diverse aspects of natural control of cyanobacterial blooms, ranging from the role of cyanophages and chytrids on cyanobacteria and the viral influence on phytoplankton community dynamics. McKindles et al. focused on understanding the diversity and role of cyanophages in bloom control in Sandusky Bay, revealing the molecular characterization of *Planktothrix* specific cyanophages. Transcriptomic analysis revealed only low levels of viral gene expression despite the highly abundant cyanophages over the course of multiple years. The study also aimed to design monitoring methods for bloom lysis events, where potentially toxins are being released. Wagner et al. explored the dynamics of chytrid (*Rhizophydium* sp.) infections in *Planktothrix agardhii* blooms, with modified environmental conditions in mesocosms to explore infection prevalence. The results identified temperature and water flow as crucial factors in chytrid prevalence. The study provides valuable insights into control measures to reduce blooms. Lastly, Peng et al. investigated viral influences on phytoplankton community succession in the Three Gorges Reservoir, emphasizing the multiple roles viruses play in summer bloom dynamics. The viral lysis of eukaryotes and increase of nutrients due to the lysis of bacterioplankton appeared to be beneficial for the success of cyanobacteria. Together, these studies provide comprehensive insights into the complex interactions shaping cyanobacterial blooms, offering valuable knowledge for developing effective control strategies and safeguarding water ecosystems.

Concluding the Research Topic, three papers present data on metabolites that include both the synthesis of the common cyanotoxin, microcystin, as well as other products that arise in concert with bloom events. Extending prior work on the temperature dependence of microcystin accumulation in *Microcystis* sp. at lower temperatures, Roy et al. showed that a wild type *Microcystis* toxin-producing strain, has a growth advantage over its Δ*mcyB* (nontoxic) mutant strain at both low (20°C) and high temperature (30 and 35°C), and that cellular quotas of soluble microcystins are lower at higher temperature. However, higher temperature yields a shift in the intracellular toxin pool. This paper helps provide a better understanding of the role of microcystins in adaptation of *Microcystis* sp. to long-term and diel temperature shifts.

The papers by Zhou et al. and Lee et al. examined other metabolites and factors associated with blooms that may also affect environmental and human health. Zhou et al. have studied exudates of *Microcystis aeruginosa* that can be inhibitory to aquatic life, and in this paper, they employed LC/MS to characterize exudates from non-toxic and toxic *Microcystis* strains during exponential and stationary phases of growth. Whereas the toxin-producing strain produced a higher diversity of exudate compounds, the non-toxic strain produced higher concentrations of potentially ecotoxic metabolites. This work demonstrated that even non-toxic bloom formers can produce compounds that degrade ecosystem health. The paper by Lee et al. raises awareness of both disinfection byproducts by water treatment of cHABs, and of potential pathogens (*Legionella* sp.) and antibiotic resistance genes associated by blooms. Evidently, bloom biomass may afford multiple threats to health which extend past the well-known consequences of microcystin exposure.

## Author contributions

GB: Writing – review & editing. RM: Writing – review & editing. PV: Writing – review & editing.

